# All-Arthroscopic Anterior and Posterior Arthrolysis for Extension Deficit After Anterior Cruciate Ligament Reconstruction

**DOI:** 10.1016/j.eats.2025.103774

**Published:** 2025-08-06

**Authors:** Mohamad K. Moussa, Victor Sonnery-Cottet, Stefano Jacotti, Mohammed Khatiri, Regis Pailhé, Alexandre Hardy, Etienne Cavaignac

**Affiliations:** a Group Hospitalier Sélestat Obernai, Obernai, France; b CHU Toulouse, Toulouse, France; c Clinique Aguiléra–Ramsay Santé, Biarritz, France; d Clinique du Sport, Paris, France

## Abstract

Persistent knee extension deficit after anterior cruciate ligament reconstruction is a disabling complication often associated with intra-articular fibrosis within the intercondylar notch and posterior capsular adhesions. This article presents a safe, efficient, and reproducible all-arthroscopic technique for treating such deficits via combined anterior and posterior arthrolysis. The procedure includes resection of cyclops lesions and anterior fibrotic tissue, followed by posterior capsular release using posteromedial, trans-septal, and posterolateral portals. In contrast to open approaches, this technique allows controlled capsular release while limiting surgical morbidity. Intraoperative assessment typically shows restoration of full extension, with reduced operative time and limited soft tissue trauma. This minimally invasive technique provides an effective option for early management of knee stiffness following anterior cruciate ligament reconstruction and warrants further validation through prospective clinical studies.

Knee stiffness following anterior cruciate ligament reconstruction (ACLR) is a significant complication that negatively impacts the functional and clinical outcomes of the surgery.^1^, ^2^, ^3^, ^4^, ^5^, ^6^, ^7^ This complication manifests primarily as an extension deficit, with or without associated flexion deficits, knee pain, and altered knee kinematics.^4^^,^^6^^,^^8^^,^^9^ Several factors have been implicated in the occurrence of extension deficits, including surgical technique, inadequate rehabilitation protocols, graft impingement, soft tissue fibrosis, arthrogenic muscle inhibition, and capsular adhesions.^4^^,^^6^^,^^8^^,^^10^ Another recognized cause is the presence of a cyclops lesion, defined as a fibrous nodule situated in the anterior intercondylar notch, restricting terminal knee extension.^2^^,^^7^^,^^11^ Posterior capsular adhesions also significantly contribute to persistent extension deficits, particularly when anterior arthrolysis alone fails to resolve the stiffness.^5^^,^^9^

Traditionally, cyclops lesions and anterior adhesions are treated via arthroscopic anterior arthrolysis,^3^^,^^12^^,^^13^ while posterior capsular adhesions are managed using various surgical approaches, ranging from open posterior capsulotomy to arthroscopic capsular release techniques.^14^, ^15^, ^16^, ^17^, ^18^, ^19^ Although open techniques provide extensive exposure, they are associated with increased procedural trauma, higher risks of neurovascular injury, and potential postoperative complications, such as recurrent scarring and stiffness.^15^^,^^18^^,^^19^ In contrast, an all-arthroscopic approach to anterior and posterior arthrolysis offers the advantages of minimally invasive surgery, including decreased postoperative pain, faster recovery, shorter operative times, and reduced risk of complications.^5^^,^^14^^,^^16^^,^^17^^,^^20^ Given these benefits, arthroscopic arthrolysis emerges as an appealing treatment modality, especially for early postoperative knee stiffness following ACLR.^5^^,^^14^^,^^16^^,^^17^^,^^20^

The purpose of this article is to present a detailed surgical technique of all-arthroscopic anterior and posterior arthrolysis for treating knee stiffness characterized by persistent extension deficit following ACLR.

## Surgical Technique

### Positioning

**Fig 1:**
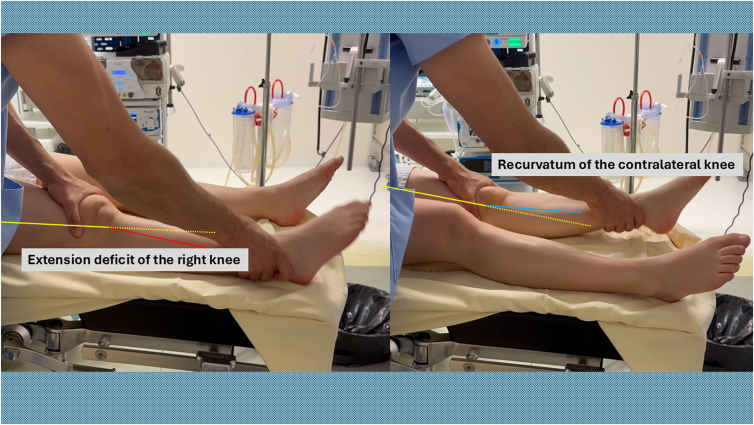
Bilateral assessment of knee extension is performed preoperatively with the patient in the supine position to document range of motion and identify asymmetry. This comparison establishes the baseline extension deficit before arthrolysis.

Begin the procedure with a bilateral assessment of knee extension to document the preoperative range of motion (Fig 1). The patient is positioned supine with the knee flexed to 90° to maximize the distance from surrounding neurovascular structures. A pneumatic tourniquet is applied high on the thigh and inflated to 300 mm Hg.

### Arthroscopic Exploration

**Fig 2:**
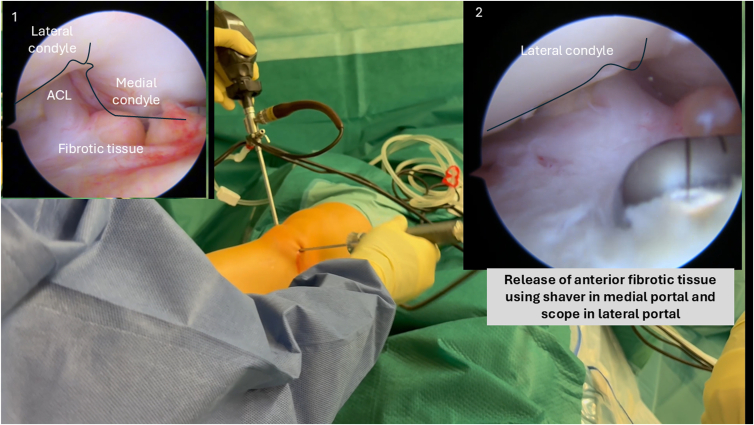
Arthroscopic view from the anterolateral portal of a right knee in 90° of flexion. Dynamic exploration of the anterior compartment is performed, followed by resection of anterior fibrotic tissue—including cyclops lesions—using a motorized shaver to improve terminal extension.

Arthroscopic exploration is performed through standard anterolateral and anteromedial portals. Perform a dynamic exploration of the anterior compartments of the knee, starting with the suprapatellar region to the medial and lateral compartment of the knee. The intercondylar notch area is closely inspected for cyclops lesions or any anterior fibrotic tissue that may impede full knee extension. Dynamic assessment involves passive flexion and extension of the knee (Fig 2).

### Anterior Arthrolysis

Using a motorized shaver (Arthrex), all cyclops tissue and impinging soft tissues are removed carefully to help achieve full extension of the knee. Adhesions involving the anterior cruciate ligament, posterior cruciate ligament, intercondylar notch, and anterior recesses are released thoroughly without notchplasty (Fig 2). We strongly advise against performing anterior arthrolysis as a standalone procedure due to the high risk of recurrence. A combination of anterior and at least posteromedial arthrolysis should always be performed to optimize outcomes.

### Posterior Arthrolysis

##### Posteromedial Portal Creation

With the knee maintained at 90° of flexion, the arthroscope introduced through the anterolateral portal is guided posteriorly between the posterior cruciate ligament and the medial femoral condyle to access the posteromedial recess and visualize the meniscal ramp.

**Fig 3:**
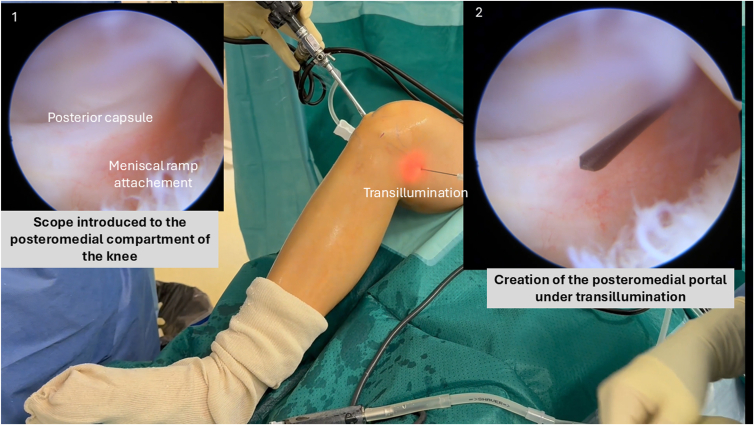
Creation of the posteromedial portal under transillumination guidance in a right knee flexed to 90°. The arthroscope is introduced through the anterolateral portal and advanced posteriorly between the posterior cruciate ligament and medial femoral condyle to visualize the posteromedial recess.

Under transillumination guidance, a posteromedial portal is safely established using a spinal needle, ensuring adequate distance from the medial femoral condyle to prevent injury to the saphenous nerve and vein (Fig 3).

##### Posteromedial Capsule Release

**Fig 4:**
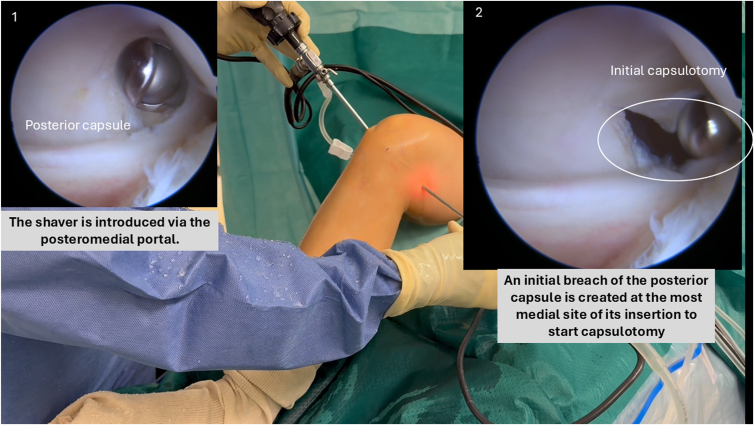
Arthroscopic view from the anterolateral portal showing the initial capsulotomy at the most medial aspect of the posterior capsule in a right knee. A motorized shaver, introduced in the posteromedial portal, is used to establish a controlled breach before progressing to full release.

A motorized shaver is introduced through the posteromedial portal, always oriented anteriorly toward the joint to protect neurovascular structures. An initial breach in the posterior capsule is created at the spinal needle insertion site (most medial part of the capsule) using the shaver, facilitating subsequent insertion of basket forceps (Fig 4).

**Fig 5:**
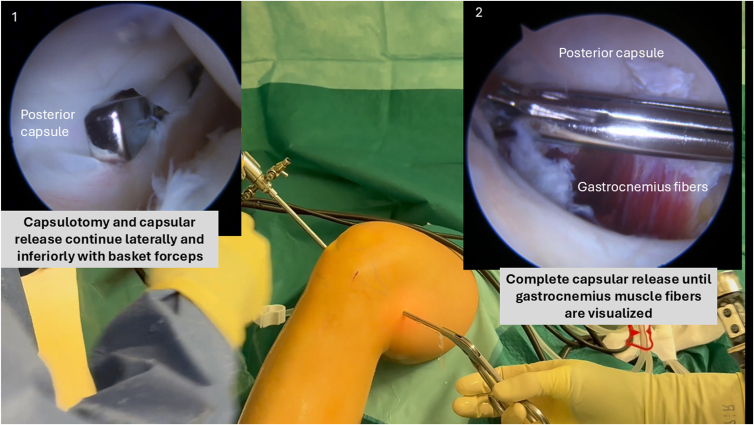
Complete posteromedial capsular release is performed using a basket forceps introduced through the posteromedial portal in a right knee. The capsule is dissected off the tibial plateau until the gastrocnemius muscle fibers are exposed.

Capsulotomy and capsular release continue laterally with basket forceps, keeping the opening of the forceps anteriorly, dissecting the capsule from the tibial plateau until gastrocnemius muscle fibers are visualized. The capsular release is extended laterally up to the central septum (Fig 5). After anterior and posteromedial arthrolysis, reassess extension. If full extension is not achieved, posterior lateral capsule should be released.

##### Trans-septal Approach

With the scope still in the anterolateral portal, the central inferior septum is visualized. It is gently excised using a shaver introduced via the posteromedial portal, carefully keeping the shaver blade facing anteriorly to protect the popliteal neurovascular structures. The arthroscope is then introduced through the posteromedial portal and then advanced through the septum.

##### Posterolateral Portal Creation

**Fig 6:**
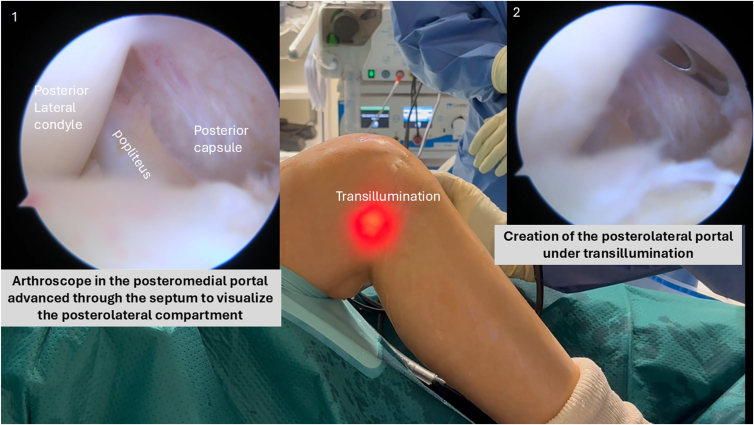
Creation of the posterolateral portal under transillumination guidance in a right knee flexed to 90°, with the arthroscope positioned in the posteromedial portal. The portal is established posterior to the lateral collateral ligament and popliteus tendon, allowing safe access to the posterolateral compartment while avoiding neurovascular structures.

Under direct arthroscopic visualization and using transillumination with spinal needle guidance, the posterolateral portal is placed posterior to the lateral collateral ligament and the popliteus tendon at the joint line (Fig 6).

##### Posterolateral Capsule Release

This stage is performed in case of persistent extension deficit despite anterior and posteromedial arthrolysis.

**Fig 7:**
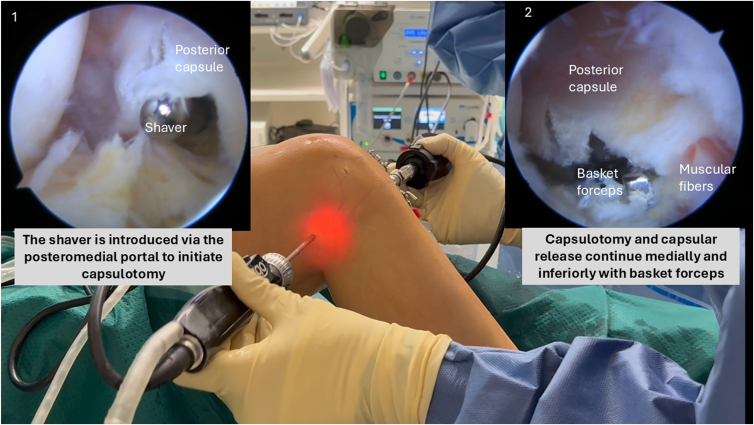
Arthroscopic view from the posteromedial portal in a right knee showing posterolateral capsular release. A motorized shaver introduced through the posterolateral portal is used to complete the capsulotomy and expose the posterolateral tibial plateau.

With the arthroscope remaining in the posteromedial portal, the motorized shaver introduced through the posterolateral portal continues to remove any residual septal tissue, fully exposing the posterolateral capsule. Similar to the posteromedial side, an initial capsular breach is created at the spinal needle insertion site (at the most lateral part of the capsule) using the shaver. The basket forceps then complete the capsulotomy and capsular release inferiorly and medially along the tibial plateau until the gastrocnemius muscle fibers are clearly visualized (Fig 7).

Closure of the arthroscopic portals is performed using resorbable sutures.

The pearls and pitfalls of the technique are presented in Table 1. The surgical video is presented in Video 1.

**Table 1:** Pearls and Pitfalls of the Technique

Pearls	Pitfalls
Use of motorized shaver and basket forceps allows precise dissection of thick adhesions.	Aggressive debridement risks iatrogenic damage to the cruciate ligaments or articular cartilage. Excessive use of thermal devices may increase the risk of soft tissue necrosis or delayed healing.
A combination of anterior and at least posteromedial arthrolysis should always be performed to optimize outcomes.	We strongly advise against performing anterior arthrolysis as a standalone procedure due to the high risk of recurrence.
After anterior and posteromedial arthrolysis, reassess extension. If full extension is not achieved, the posterior lateral capsule should be released.	Incomplete release of the posterior capsule may lead to residual extension deficit.
Posteromedial and posterolateral portals enhance safety and visualization of critical structures.	Poor portal placement may increase the risk of neurovascular injury, especially in posterior compartments.
Maintain intraoperative full range-of-motion testing after each compartment is addressed.	

### Postoperative Protocol

Early rehabilitation is initiated to maintain the gained extension. Initial therapy prioritizes passive and active knee mobilization exercises, emphasizing full extension. Early quadriceps activation exercises, including isometric quadriceps sets and neuromuscular electrical stimulation, are recommended. Full weightbearing is allowed according to patient comfort and progress, generally within the first postoperative weeks.

Pain management is provided with analgesics and anti-inflammatory medications as needed.

## Discussion

This article presents a safe, efficient, and reproducible all-arthroscopic technique for anterior and posterior arthrolysis in the treatment of postoperative knee extension deficits. Loss of knee extension following ACLR significantly impairs functional outcomes, posing substantial challenges for surgeons and patients alike.^1^, ^2^, ^3^, ^4^, ^5^, ^6^, ^7^ Historically, open posterior capsulotomy provided direct access and visualization but resulted in greater morbidity, longer recovery, and increased risk of neurovascular complications.^15^ As minimally invasive techniques evolved, arthroscopic methods gained preference due to reduced surgical trauma, superior visualization, faster rehabilitation, and lower complication rates.^5^^,^^14^^,^^16^^,^^17^^,^^20^

Several arthroscopic approaches to posterior arthrolysis have been described, showing excellent results for the treatment of recalcitrant flexion contractures.^5^^,^^14^^,^^16^^,^^17^^,^^20^ For instance, Reinholz et al.^16^ and Brinkman et al.^14^ reported effective restoration of terminal knee extension through arthroscopic posterior capsular releases, achieving significant improvements in patient-reported outcomes. Meanwhile, Kylies et al.^9^ presented an extensive arthroscopic arthrolysis method systematically addressing adhesions within multiple knee compartments, including a trans-septal posterior approach.

Suresh et al.^17^ introduced a percutaneous spinal needle technique, enhancing minimal invasiveness and reducing operative morbidity through careful layer-by-layer posterior capsule sectioning. Unlike Suresh et al.,^17^ the current technique uses conventional arthroscopic instrumentation (motorized shavers and basket forceps) to meticulously dissect and remove thicker, more robust capsular adhesions. Although the spinal needle approach is minimally invasive and carries a low-risk profile, its effectiveness may be limited when encountering dense and extensive fibrosis. Conversely, our technique enables adequate capsular release and efficient removal of thicker scar tissue, allowing greater depth control and tactile feedback during dissection. Furthermore, our technique benefits from recent advances in posterior knee arthroscopy, specifically employing dedicated posteromedial, trans-septal, and posterolateral portals to achieve controlled, extensive, and precise posterior capsular release, enhancing safety and precision compared to single-portal or purely percutaneous techniques.^9^^,^^21^, ^22^, ^23^, ^24^ Additionally, the short surgical time and limited postoperative edema may contribute to faster rehabilitation and reduced hospital stay as compared to open techniques. However, the technique has limitations. It requires advanced arthroscopic skills, particularly in navigating the posterior compartments and managing the trans-septal approach. Incomplete portal placement or dissection may increase the risk of neurovascular injury, particularly in the posterior capsule. Dense extra-articular fibrosis cannot be addressed arthroscopically and may require conversion to an open approach (Table 2).

**Table 2:** Advantages and Limitations of the Technique

Advantages	Limitations/Risks
Minimally invasive approach with reduced morbidity compared to open surgery	Requires advanced arthroscopic skills and familiarity with posterior compartment anatomy
Comprehensive capsular release via posteromedial, trans-septal, and posterolateral portals	Risk of neurovascular injury if portals are mispositioned or dissection is uncontrolled
Enhanced visualization of posterior compartments under direct arthroscopic control	Extra-articular fibrosis may not be accessible arthroscopically
Shorter surgical time and limited soft tissue trauma	

In our clinical experience, this approach consistently achieves immediate postoperative restoration of full knee extension during intraoperative assessment, benefiting from shorter surgical duration and reduced postoperative edema. An essential prerequisite for arthroscopic arthrolysis is a competent quadriceps and vastus medialis function; otherwise, the risk of recurrence is significantly increased. Moreover, no strict temporal cutoff post-ACLR restricts the indication; rather, intervention is advised whenever a fixed flexion deformity is confirmed alongside functional quadriceps muscle activation. However, clinical outcomes require formal evaluation through prospective cohort studies before broader recommendations can be made, despite growing evidence supporting the efficacy of all-arthroscopic methods.^5^^,^^14^^,^^16^^,^^17^^,^^20^

Nonetheless, severe extra-articular adhesions or extensive fibrosis involving periarticular structures (e.g., patellar tendon or iliotibial band) may not be fully addressed arthroscopically, sometimes necessitating complementary open surgical procedures.

## Disclosures

The authors declare the following financial interests/personal relationships which may be considered as potential competing interests: A.H. is a consultant or advisor for Arthrex and Depuy. E.C. is a consultant or advisor for Arthrex and Amplitude. All other authors (M.K.M., V.S-C., S.J., M.K., R.P.) declare that they have no known competing financial interests or personal relationships that could have appeared to influence the work reported in this paper.

## Supplementary Data

Video 1 This surgical video shows a systematic, all-arthroscopic anterior and posterior arthrolysis technique for treating persistent knee extension deficit following anterior cruciate ligament reconstruction. The procedure is performed on the right knee with the patient in the supine position and the knee flexed to 90°. Step-by-step operative sequences include preoperative bilateral extension testing, anterior arthrolysis addressing cyclops and fibrotic tissue through anteromedial and anterolateral portals, and posterior capsular release via posteromedial, trans-septal, and posterolateral approaches. Portal placement, safe instrumentation, and key anatomic landmarks are illustrated to enhance procedural safety and efficacy. The video concludes with confirmation of full intraoperative knee extension.
